# Monoamine oxidase‐A is a novel driver of stress‐induced premature senescence through inhibition of parkin‐mediated mitophagy

**DOI:** 10.1111/acel.12811

**Published:** 2018-07-12

**Authors:** Nicola Manzella, Yohan Santin, Damien Maggiorani, Hélène Martini, Victorine Douin‐Echinard, Joao F. Passos, Frank Lezoualc'h, Claudia Binda, Angelo Parini, Jeanne Mialet‐Perez

**Affiliations:** ^1^ Institute of Metabolic and Cardiovascular Diseases (I2MC) Institut National de la Santé et de la Recherche Médicale (INSERM), Université de Toulouse Toulouse France; ^2^ Department of Biology and Biotechnology University of Pavia Pavia Italy; ^3^ Ageing Research Laboratories Newcastle University Institute for Ageing, Newcastle University Newcastle upon Tyne UK

**Keywords:** cardiac, mitochondria, mitophagy, monoamine oxidase, oxidative stress, senescence

## Abstract

Cellular senescence, the irreversible cell cycle arrest observed in somatic cells, is an important driver of age‐associated diseases. Mitochondria have been implicated in the process of senescence, primarily because they are both sources and targets of reactive oxygen species (ROS). In the heart, oxidative stress contributes to pathological cardiac ageing, but the mechanisms underlying ROS production are still not completely understood. The mitochondrial enzyme monoamine oxidase‐A (MAO‐A) is a relevant source of ROS in the heart through the formation of H_2_O_2_ derived from the degradation of its main substrates, norepinephrine (NE) and serotonin. However, the potential link between MAO‐A and senescence has not been previously investigated. Using cardiomyoblasts and primary cardiomyocytes, we demonstrate that chronic MAO‐A activation mediated by synthetic (tyramine) and physiological (NE) substrates induces ROS‐dependent DNA damage response, activation of cyclin‐dependent kinase inhibitors p21^cip^, p16^ink4a^, and p15^ink4b^ and typical features of senescence such as cell flattening and SA‐β‐gal activity. Moreover, we observe that ROS produced by MAO‐A lead to the accumulation of p53 in the cytosol where it inhibits parkin, an important regulator of mitophagy, resulting in mitochondrial dysfunction. Additionally, we show that the mTOR kinase contributes to mitophagy dysfunction by enhancing p53 cytoplasmic accumulation. Importantly, restoration of mitophagy, either by overexpression of parkin or inhibition of mTOR, prevents mitochondrial dysfunction and induction of senescence. Altogether, our data demonstrate a novel link between MAO‐A and senescence in cardiomyocytes and provides mechanistic insights into the potential role of MAO‐dependent oxidative stress in age‐related pathologies.

## 1 INTRODUCTION

Cell senescence is a process that is characterized by irreversible cell‐cycle arrest, resistance to apoptosis and a dramatic phenotypic remodelling. Senescent cells have also been shown to secrete several pro‐inflammatory factors, collectively known as the senescence‐associated secretory phenotype (Childs, Durik, Baker, & van Deursen, 2015). Compelling evidence indicates that chronic accumulation of senescent cells is deleterious during the ageing process and has been causally implicated in the functional decline of organs, such as kidney, heart and liver (Baker et al., 2016; Zhu et al., 2015).

One of the central drivers of senescence is the DNA damage response (DDR), which initiates the activation of the p53/p21^cip^ and p16^ink4A^ pathways, two major contributors to cell cycle arrest (Childs et al., 2015). Cellular senescence is classically thought to be the result of telomere shortening, which occurs as a consequence of replicative exhaustion. However, various other stressors, such as oxidative stress, have been shown to induce cellular senescence prematurely, a process commonly referred to as stress‐induced premature senescence (SIPS).

Mitochondria are both sources and targets of reactive oxygen species (ROS), and mitochondrial dysfunction has been considered a hallmark of pathological ageing (Lopez‐Otin, Blasco, Partridge, Serrano, & Kroemer, 2013). At the cellular level, converging evidence indicates that mitochondrial dysfunction and cellular senescence are interlinked processes (Ziegler, Wiley, & Velarde, 2015). This is well‐illustrated by the finding that complete removal of mitochondria from senescent cells prevents several features of cellular senescence (Correia‐Melo et al., 2016). Furthermore, during senescence, the mTOR/p70S6K kinase pathway has been shown to induce mitochondrial dysfunction and enhance ROS production, which stabilizes the DDR in a positive feedback loop (Correia‐Melo et al., 2016; Nacarelli, Azar, & Sell, 2015). Currently, it remains unclear why damaged mitochondria accumulate in senescent cells, although it may involve increased biogenesis and/or disruption of mitophagy, a process which allows their elimination by the autophagy‐lysosome pathway (Korolchuk, Miwa, Carroll, & von Zglinicki, 2017). In addition, there is a strong need to ascertain the sources of mitochondrial ROS that trigger premature senescence in order to target age‐associated disorders.

Monoamine oxidase‐A (MAO‐A) is a mitochondrial FAD‐dependent enzyme that catalyses the oxidative deamination of serotonin and catecholamines and generates hydrogen peroxide (H_2_O_2_) as a by‐product of the enzymatic reaction. In the heart, MAO‐A has been demonstrated to be an important source of oxidative stress in acute and chronic pathological conditions (Bianchi et al., 2005; Kaludercic et al., 2010). In particular, cardiac overexpression of MAO‐A drives oxidative stress and mitochondrial damage, leading to cell death and heart failure (Kaludercic, Mialet‐Perez, Paolocci, Parini, & Di Lisa, 2014; Villeneuve et al., 2013). Notably, all these effects are observed with high levels of MAO‐A activation, and it is well‐established that depending on its concentration, H_2_O_2_ can have multiple dose‐dependent cellular responses, ranging from proliferation to necrosis (Giorgio, Trinei, Migliaccio, & Pelicci, 2007). p53 is one of the main effectors of the MAO‐A/H_2_O_2_ axis (Villeneuve et al., 2013), which is at the crossroad of numerous signalling pathways and, depending on the extent of damage, can initiate DNA repair, senescence or cell death (Green & Kroemer, 2009). These observations, together with the fact that MAO‐A expression increases in the ageing heart (Maurel et al., 2003), led us to investigate whether a chronic increase in H_2_O_2_ through persistent MAO‐A activation could drive senescence in cardiac cells.

Here, we demonstrate that MAO‐driven ROS production at sublethal doses induces a DDR and a senescence phenotype that is characterized by increased expression of p21, p15 and p16, increased SA‐β‐gal activity and cell enlargement. Importantly, our data indicate that MAO‐A activation leads to the accumulation of dysfunctional mitochondria through the disruption of mitophagy, which is mediated by p53‐induced parkin inhibition. Moreover, we show that the mTOR kinase pathway contributes to mitophagy dysfunction by enhancing cytosolic p53 accumulation. Finally, our results indicate that inhibition of mitophagy and the resulting accumulation of damaged mitochondria can explain all the features of the senescent phenotype induced by MAO‐A activity.

## 2 RESULTS

### 2.1 MAO‐A activity increases during ageing and triggers oxidative stress‐mediated DNA damage response

**Figure 1:**
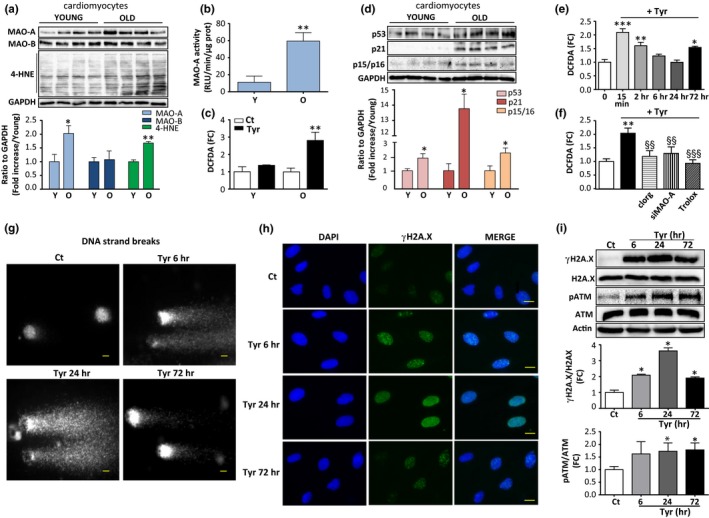
Monoamine oxidase‐A (MAO‐A) and oxidative stress are increased in senescent mouse cardiomyocytes, and stimulation of MAO‐A triggers reactive oxygen species (ROS)‐dependent DNA damage response (DDR) in H9C2 cells. (a) Immunoblots of MAO‐A, MAO‐B and 4‐HNE protein adducts in adult ventricular myocytes from young (3 months) and old (20 months) mice. Quantifications of the ratios to GAPDH are shown in the lower panel (*N* = 4). (b) MAO‐A activity in isolated cardiomyocytes from young (3 months) and old (20 months) mice (*N* = 4). (c) MAO‐A‐mediated DCFDA oxidation in response to 500 μM Tyr for 2 hr in adult ventricular myocytes from young (3 months) and old (20 months) mice (*N* = 4). (d) Immunoblots of p53, p21 and p15/p16 proteins in adult ventricular myocytes from young (3 months) and old mice (20 months) mice. Quantifications of the ratios to GAPDH are shown in the lower panel (*N* = 4). (e) Time‐course of DCFDA oxidation in response to 500 μM Tyr in H9C2 cells (*N* = 5). (f) DCFDA oxidation in response to 500 μM Tyr for 15 min alone or in the presence of clorg (10 μM), siMAO‐A siRNA or Trolox (500 μM) in H9C2 (*N* = 4). (g) Comet assay representing DNA damage (DNA strand breaks) in H9C2 cells treated with Tyr (500 μM) for the indicated time. Scale Bar = 10 μm. Images are representative of *N* = 3 experiments. (h) DNA damage foci (reflected by γH2A.X foci) in H9C2 treated with 500 μM Tyr for the indicated times. DAPI‐labelled nuclei are in blue and γH2A.X is stained in green. Scale Bar = 10 μm. Images are representative of *N* = 3 experiments. (i) Immunoblots of total and phosphorylated levels of H2A.X and ATM in H9C2 cells stimulated with Tyr (500 μM) for the indicated time. Actin was used as a loading control. Quantifications of the ratios of γH2A.X to total H2A.X and phospho‐ATM to total ATM are shown in the lower panels (*N* = 4). Data are expressed as the mean ± *SEM* (*p < 0.05, **p < 0.01, ***p < 0.001 vs. young mice or control; §§*p* < 0.01, §§§*p* < 0.001 vs. Tyr)

We first analysed the effects of ageing on MAO expression, oxidative stress and senescent markers in adult mouse ventricular myocytes. MAO‐A, but not MAO‐B, was upregulated in 20‐month‐old compared to 3‐month‐old cardiomyocytes together with 4‐hydroxynonenal (4‐HNE), a marker of lipid peroxidation (Figure 1a). An increase in MAO‐A enzymatic activity was observed in old cardiomyocytes paralleled by an increase in ROS generation in response to the MAO substrate tyramine (Tyr) (Figure 1b,c). Classical senescent markers, such as p53, p21 and p15/p16, were also significantly elevated in aged cardiomyocytes at the protein level (Figure 1d). We next evaluated the putative causal link between enhanced MAO‐A activity, ROS and senescent markers in H9C2 cells that endogenously express MAO‐A but not MAO‐B. A time‐course of Tyr stimulation demonstrated a rapid increase in the oxidation of the fluorescent probe DCFDA, which was maximal at 15 min and decreased progressively to the baseline levels at 24 hr (Figure 1e). This increase in ROS production was in the same range as the one observed in old cardiomyocytes stimulated with Tyr (Figure 1c). ROS generation induced by Tyr was prevented by the selective MAO‐A inhibitor clorgyline (clorg), by siRNA mediated knockdown of MAO‐A (siMAO‐A, Figure S1a) or treatment with the antioxidant Trolox (Figure 1f). H_2_O_2_ is the main product resulting from the catalytic activity of MAO‐A. The extracellular H_2_O_2_ concentration was significantly elevated 1 hr after Tyr stimulation, and prior application of clorg‐, Trolox‐ or siRNA‐mediated knockdown of MAO‐A prevented this effect (Figure S1b). Notably, at the concentration used, Tyr incubated for 24 hr did not induce cytotoxic effects (Figure S1c,d).

To evaluate whether MAO‐A‐induced oxidative stress led to DNA damage and the consequent activation of the DDR, levels of DNA damage were assessed by the comet assay over a 72 hr period of Tyr stimulation. The results showed a rapid and persistent increase in DNA strand breaks from 6 to 72 hr after Tyr stimulation (Figure 1g). The DDR is characterized by the activation of ataxia‐telangiectasia mutated kinase (ATM) and the formation of DNA‐damage foci containing a phosphorylated form of H2A.X (γH2A.X). Consistent with an activation of the DDR, immunofluorescence assays revealed the presence of increased nuclear γH2A.X foci in cells stimulated with Tyr from 6 to 72 hr (Figure 1h and Figure S1e). In line with these findings, an acute and persistent activation of the DDR was confirmed by a significant increase in the phosphorylation levels of ATM and H2A.X in immunoblots (Figure 1i). Altogether, these observations demonstrate that chronic MAO‐A activation at sublethal doses results in oxidative stress, DNA strand breaks and a persistent DDR.

### 2.2 MAO‐A stimulation promotes cyclin‐dependent kinase inhibitor (CDKi) activation and SIPS in H9C2 and primary cardiomyocytes

**Figure 2:**
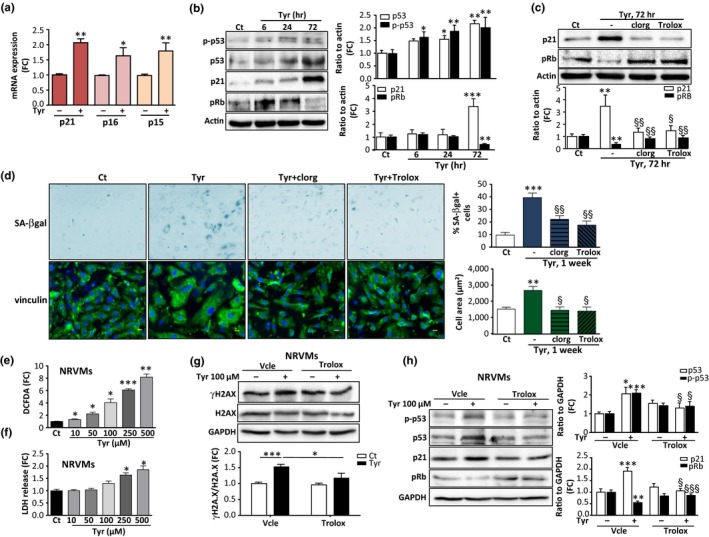
Monoamine oxidase‐A (MAO‐A) activation induces expression of cell cycle inhibitors and senescence markers in H9C2 and primary cardiomyocytes. (a) Analysis of mRNA levels of CDKi p21^cip^, p16^ink4a^ and p15^ink4b ^normalized to GAPDH by real‐time RT‐PCR in H9C2 cells stimulated with 500 μM Tyr for 72 hr (*N* = 4). (b) Immunoblots of phospho‐Ser15‐p53, total p53, p21 and phospho‐Rb in H9C2 stimulated with 500 μM Tyr for the indicated times. Quantifications of the ratios to actin are shown on the histograms (right panels) (*N* = 3). (c) Immunoblots of p21 and pRb in cells stimulated with 500 μM Tyr for 72 hr in control conditions or in the presence of clorg (10 μM) or Trolox (500 μM), when indicated. Quantifications of the ratios to actin are shown on the histogram below (*N* = 3). (d) Representative images and quantitative analysis of the percent of SA‐β‐gal + cells (blue staining) and cell area (vinculin staining in green) after stimulation with 500 μM Tyr for 1 week in the presence of clorg (10 μM) or Trolox (500 μM), when indicated. The number of blue cells positive for SA‐β‐gal was expressed as % of total cell number (100 cells counted for each condition, *N* = 4 independent experiments, Scale Bar = 100 μm). For cell area, DAPI (blue) was used to label nuclei and vinculin was used for cell size measurement (100 cells were counted for each condition, *N* = 4 independent experiments, Scale Bar = 10 μm). (e) Neonatal rat ventricular myocytes (NRVMs) transduced with MAO‐A adenovirus were stimulated with increasing concentrations of Tyr for 6 hr to measure DCFDA oxidation (*N* = 4). (f) Neonatal rat ventricular myocytes (NRVMs) transduced with MAO‐A adenovirus were stimulated with increasing concentrations of Tyr for 72 hr to measure cytotoxicity by LDH release (*N* = 4). (g) Immunoblots of γH2A.X and total H2A.X in neonatal rat ventricular myocytes (NRVMs) transduced with MAO‐A adenovirus and stimulated with 100 μM of Tyr for 72 hr. Quantifications of the ratios to GAPDH are shown on the histogram below (*N* = 6). (h) Immunoblots of p‐p53, p53, p21 and pRb in neonatal rat ventricular myocytes (NRVMs) transduced with MAO‐A adenovirus and stimulated with 100 μM of Tyr for 72 hr. Quantifications of the ratios to GAPDH are shown in the right histograms (*N* = 6). Data are expressed as the mean ± *SEM* (*p < 0.05, **p < 0.01, *** *p* < 0.001 vs. control; §*p* < 0.05, §§*p* < 0.01, §§§*p* < 0.001 vs. Tyr)

Several studies have shown that the DDR is the main trigger of senescence and leads to the downstream activation of CDKi of the p53/p21^cip^ or Ink4 family (p15^ink4b^, p16^ink4A^) (Campisi & d'Adda di Fagagna, 2007). These CDKi maintain the retinoblastoma protein (Rb) in a hypo‐phosphorylated state, preventing the progression of the cell cycle. As shown in Figure 2a, relative mRNA expression of the classical CDKi p21^cip^, p16^ink4A^ and p15^ink4b^ was increased 72 hr after Tyr treatment. In addition, we observed an increase in p53 phosphorylation (p‐p53) at Ser15, an increase in total p53 and its downstream target p21, and a decrease in Rb phosphorylation (pRb) (Figure 2b). Clorg and Trolox prevented Tyr‐induced p21 expression and Rb dephosphorylation, confirming the specific role of MAO‐A in this pathway (Figure 2c). The senescence phenotype has been characterized by increased activity of β‐galactosidase at pH 6, referred to as senescence‐associated β‐galactosidase (SA‐β‐gal). At 7 days post‐Tyr exposure, the number of SA‐β‐gal‐positive cells was significantly raised (Figure 2d). In addition, cells became flattened and showed a significant increase in cellular area, which are also characteristics of senescence (Figure 2d). MAO‐A inhibition with clorg or ROS scavenging with Trolox significantly reduced these senescent phenotypes, as shown by a decrease in the percent of SA‐β‐gal‐positive cells and a decrease in the cellular area (Figure 2d). Finally, we measured the proliferative potential of cells 1 week after Tyr treatment. We found that the rate of proliferation was reduced with Tyr compared to untreated cells, and prior treatment with clorg and Trolox inhibited this effect (Figure S1f).

Next, we evaluated whether the MAO‐A/ROS/DDR/senescence pathway could be activated by the endogenous MAO substrate norepinephrine (NE), which is known to exert many of its effects through adrenergic receptors in cardiac cells. Treatment of H9C2 cells with 100 µM NE did not impair cell viability but increased levels of oxidative stress, which was prevented by clorg, siRNA‐mediated MAO‐A knockdown and Trolox (Figure S2a,b). As observed with Tyr, NE induced persistent activation of the DDR (Figure S2c). Furthermore, it increased mRNA levels of p21^cip^, p16^ink4A^ and p15^ink4b^, increased protein levels of phospho(Ser15)‐p53 and p21 and decreased pRB (Figure S2d,e). Clorg and Trolox treatment reduced p21 and increased pRb expression (Figure S2f). Finally, chronic treatment with NE (1 week) increased the frequency of SA‐β‐gal‐positive cells and the mean cell area, which were reduced by clorg and Trolox (Figure S2g). The cell proliferation rate was decreased in the presence of NE, an effect which was significantly reduced by clorg and Trolox (Figure S2h). Altogether, our findings demonstrate that MAO‐A plays a significant role in the establishment of premature senescence induced by Tyr and NE in H9C2 cells.

Finally, we investigated whether the same findings could be recapitulated in primary cultures of neonatal cardiomyocytes. To drive MAO‐A expression in these cells, we used an adenovirus carrying MAO‐A cDNA (Santin et al., 2016). Tyr treatment induced a dose‐dependent increase in ROS production in cardiomyocytes (Figure 2e). On one hand, we found that above the concentration of 250 µM of Tyr, primary cardiomyocytes underwent cell death, consistent with our previous findings (Villeneuve et al., 2013) (Figure 2f). On the other hand, at doses of 100, 50 and 10 µM, no cytotoxicity was observed with Tyr up to 72 hr (Figure 2f). Interestingly, at the sublethal dose of 100 µM, chronic stimulation with Tyr for 72 hr activated the DDR, as shown by an increase in γH2A.X (Figure 2g). This effect was mediated by ROS, as Trolox inhibited H2A.X phosphorylation (Figure 2g). In addition, chronic MAO‐A activation increased the p53‐p21 pathway in cardiomyocytes and inhibited pRb, which could be prevented by Trolox (Figure 2h).

### 2.3 MAO‐A‐induced cellular senescence is associated with mitochondrial dysfunction and inhibition of parkin‐mediated mitophagy

**Figure 3:**
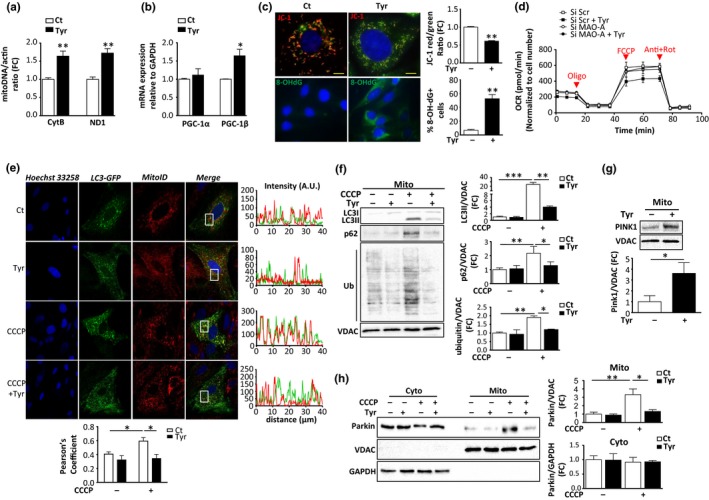
Monoamine oxidase‐A (MAO‐A) activation induces mitochondrial dysfunction and mitophagy impairment. H9C2 cells were stimulated with 500 μM Tyr for 72 hr. (a) mtDNA copy number analysis (Nd1/actin and Cytb/actin) by real‐time PCR (*N* = 4). (b) Analysis of mRNA levels of PGC‐1α and pGC‐1β normalized to GAPDH by real‐time RT‐PCR (*N* = 4). (c) *Upper panels:* JC‐1 aggregates (red)/monomers (green) for mitochondrial membrane potential and quantifications of JC‐1 red/green ratios (*N* = 4); *lower panels*: mito8‐OH‐dG immunostaining and quantification of % positive cells (*N* = 4). (d) Oxygen consumption rate (OCR) measurements in H9C2 cells transfected for 24 hr with siScr or siMAO‐A, then treated for 24 hr with Tyr and recorded 72 hr later. Recording of respiration was done at baseline and after successive addition of oligomycin, FCCP and antimycin A + rotenone. (e) Representative confocal images of LC3‐EGFP‐transfected cells stained with MitoID Red. Cells were treated with 500 μM Tyr for 72 hr, followed by 50 μM CCCP treatment for 6 hr, when indicated. The Pearson's coefficient indexes between LC3‐GFP and mitoID red fluorescence intensities were determined in 10 or more cells from three independent experiments. (f) Analysis of LC3, p62 and ubiquitinated proteins in mitochondrial extracts of H9C2 stimulated with 500 μM Tyr for 72 hr, followed by 50 μM CCCP, when indicated. VDAC was used as a loading control for mitochondria (*N* = 3). (g) Analysis of pink‐1 by immunoblot in mitochondrial extracts of H9C2 stimulated with 500 μM Tyr for 72 hr. VDAC was used as a loading control (*N* = 3). (h) Analysis of parkin by immunoblot in cytosolic and mitochondrial extracts of H9C2 cells stimulated with 500 μM Tyr for 72 hr, followed by 50 μM CCCP treatment for 6 hr, when indicated. GAPDH and VDAC were used as loading controls for the cytosolic and mitochondrial fractions, respectively (*N* = 4). Data are expressed as the mean ± *SEM* (*p < 0.05, **p < 0.01, ***p < 0.001)

Accumulation of dysfunctional mitochondria has been hypothesized to play a major role in stress‐induced senescence (Passos et al., 2010). As MAO‐A is bound to the mitochondrial outer membrane, we evaluated mitochondrial dynamics and function after cell treatment with Tyr for 72 hr. The mitochondrial mass increased following Tyr stimulation, as shown by the mitochondrial DNA copy number using mitoCytb and mitoNd1 (Figure 3a). Since previous studies have shown that increased mitochondrial biogenesis was part of the senescent phenotype, we measured the expression levels of its main transcriptional co‐activators, PGC‐1α and PGC‐1β (Correia‐Melo et al., 2016). mRNA analysis showed that PGC‐1β expression was increased with Tyr, while PGC‐1α was unchanged (Figure 3b). A drop in the mitochondrial membrane potential measured with a JC‐1 probe was seen at 72 hr after Tyr treatment in H9C2 cells (Figure 3c) and primary cardiomyocytes (Figure S3b). Furthermore, the levels of mitochondrial ROS were increased, as shown by MitoSOX fluorescence staining (Figure S3a) and the increase in mito8‐OH‐dG‐positive cells, which is indicative of mtDNA oxidation (Figure 3c). To assess mitochondrial respiration, we measured the oxygen consumption rate (OCR) in siScr‐ or siMAO‐A‐transfected cells (Figure 3d). We observed a decrease in OCR under baseline conditions after MAO‐A stimulation by Tyr (Figure S3c). Interestingly, upon addition of the ATP synthase inhibitor oligomycin, OCR decreased in Tyr‐stimulated cells, indicating that the ATP production rate was affected by MAO‐A activation. To determine maximal respiration, we then added carbonyl cyanide‐4‐(trifluoromethoxy)phenylhydrazone (FCCP), a mitochondrial uncoupler of oxidative phosphorylation. The FCCP‐stimulated increase in OCR was impaired in Tyr‐treated cells (Figure S3c). Interestingly, MAO‐A silencing with siRNA prevented the previous features, confirming the deleterious effects of MAO‐A in mitochondrial function (Figure 3d and Figure S3c). From these results, we inferred that chronic MAO‐A activation may result in the accumulation of dysfunctional and damaged mitochondria.

Mitochondrial integrity is maintained by the selective elimination of dysfunctional mitochondria with engulfment into autophagosomes and degradation by the lysosomal compartment, a process termed mitophagy (Twig, Hyde, & Shirihai, 2008). Here, we investigated whether Tyr treatment led to any changes in mitophagy using immunofluorescence staining of the autophagosome marker EGFP‐LC3 (green) and the mitochondrial marker MitoID (red). After 72 hr of Tyr treatment, we could not detect any colocalization of mitochondria with the autophagosome marker LC3 (Figure 3e). In addition, while treatment with CCCP (a well‐known inducer of mitophagy) clearly induced colocalization of mitochondria with LC3 (yellow dots), Tyr reduced this induction of mitophagy by CCCP (Figure 3e). To confirm these findings, we analysed the subcellular localization of LC3II, p62 and ubiquitinated proteins under different conditions of stimulation. As expected, CCCP strongly increased the expression of LC3II, p62 and ubiquitinated proteins in mitochondrial fractions (Figure 3f). However, Tyr alone failed to promote mitochondrial translocation of LC3 and p62, and it blocked the effects of CCCP on the accumulation of LC3II, p62 and ubiquitinated proteins in the mitochondrial fractions (Figure 3f). This inhibitory effect of MAO‐A on mitophagy was not due to a reduction of the total levels of LC3II and p62 (Figure S3d). In addition, mitophagy impairment seemed to be a characteristic feature of cardiac ageing, as demonstrated by the reduced mitochondrial/cytoplasmic ratios of parkin and p62, but not LC3II, in old hearts after mitochondrial fractionation (Figure S4a).

One of the main mechanisms of clearance of damaged mitochondria involves the pink1‐parkin‐dependent pathway, which allows recruitment of the autophagosome machinery to the mitochondria (Vincow et al., 2013). Tyr treatment resulted in the stabilization of pink1 at the mitochondria, which is generally a consequence of a drop in mitochondrial membrane potential (Figure 3g). Next, pink1 allows the recruitment of the E3 ubiquitin ligase parkin to the mitochondria, promoting ubiquitination and targeting to autophagic vesicles. We observed that treatment with CCCP stimulated the translocation of parkin from the cytoplasm to the mitochondria (Figure 3h). Tyr failed to induce parkin translocation under baseline conditions and strongly prevented CCCP‐induced translocation of parkin to the mitochondria (Figure 3h). These effects were not due to the downregulation of the total levels of parkin in Tyr‐treated cells (Figure S3e). Therefore, chronic MAO‐A stimulation inhibits parkin translocation and mitophagy, and thus may aggravate ROS‐induced mitochondrial damage by preventing the clearance of dysfunctional mitochondria.

### 2.4 Mitophagy restoration prevents mitochondrial dysfunction and abolishes the MAO‐A‐induced senescent phenotype

**Figure 4:**
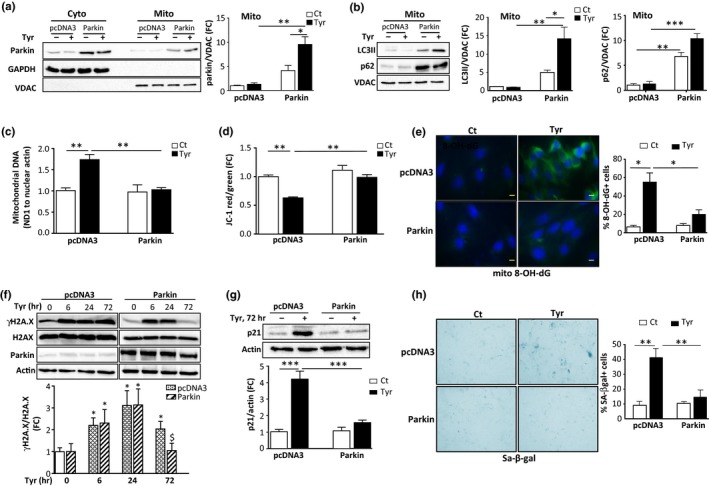
Restauration of parkin‐mediated mitophagy prevents induction of senescence. H9C2 cells were transfected with pcDNA3 or parkin and stimulated with 500 μM Tyr for 72 hr. (a) Analysis of parkin by immunoblot in cytosolic and mitochondrial extracts. GAPDH and VDAC were used as loading controls for the cyto and mito fractions, respectively (*N* = 3). (b) Analysis of LC3 and p62 by immunoblot in mitochondrial extracts. VDAC was used as a loading control (*N* = 3). (c) mtDNA copy number (Nd1/actin) by real‐time PCR (*N* = 3). (d) Quantitative analysis of JC‐1 aggregates (red)/monomer (green) ratios for mitochondrial membrane potential (*N* = 3). (e) Representative images of mito8‐OH‐dG immunostaining and quantitative analysis of 8‐OH‐dG^+^ cells as % of total cells (*N* = 3). (f) Analysis of total and γH2A.X by immunoblot in cells stimulated with Tyr for the indicated times. Actin was used as a loading control (*N* = 3). (g) Analysis of p21 expression. Actin was used as a loading control (*N* = 3). (h) Representative images and quantitative analysis of SA‐β‐gal^+^ cells as % of total cells after stimulation of the cells with Tyr for 1 week (*N* = 3). Data are expressed as the mean ± *SEM* (*p < 0.05, **p < 0.01, ***p < 0.001)

To investigate whether restoration of mitophagy impacts MAO‐A‐induced senescence, we overexpressed parkin in cardiac cells. Transfected parkin increased the total protein level of parkin (Figure S4b) that was distributed both in cytoplasm and mitochondria (Figure 4a and Figure S4c). Importantly, parkin overexpression restored mitophagy, since its transfection was associated with the enhanced mitochondrial expression of LC3II and p62 under both basal and Tyr‐treated conditions (Figure 4b).

Overexpression of parkin resulted in significant improvements in mitochondrial dynamics and function following Tyr treatment, as shown by a decreased mitochondrial mass, preservation of mitochondrial membrane potential and inhibition of mitochondrial ROS compared with pcDNA3‐transfected cells (Figure 4c–e and Figure S4d).

Subsequently, we investigated the consequences of parkin overexpression on the induction of senescence. We found that parkin overexpression did not influence the expression of γH2A.X at 6 and 24 hr after Tyr treatment, but inhibited its long‐term persistence at 72 hr (Figure 4f). Consistently, a strong decrease in p21 expression levels and SA‐β‐gal staining was observed in parkin‐transfected cells compared with pcDNA3‐transfected cells after Tyr stimulation (Figure 4g,h). Notably, we found that this effect was not due to a decrease in MAO‐A expression (Figure S4e).

In summary, our data suggest that mitochondrial dysfunction and chronic oxidative stress induced by impaired mitophagy are the mechanisms responsible for induction of cellular senescence driven by MAO‐A.

### 2.5 Crosstalk between p53 and mTOR pathways regulate mitochondrial dysfunction‐associated senescence induced by MAO‐A

**Figure 5:**
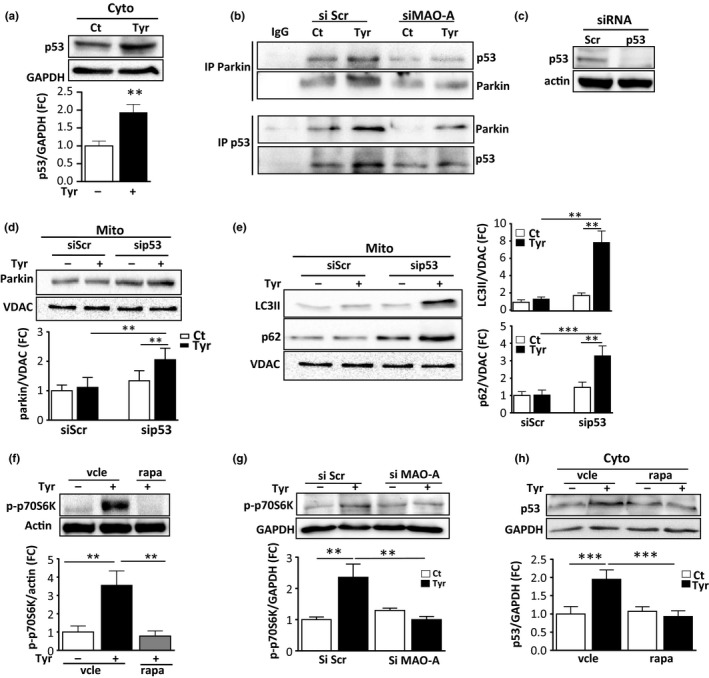
Crosstalk between mTOR and p53 in parkin‐mediated mitophagy deficiency. (a) Analysis of p53 expression in cytosolic extracts of H9C2 cells stimulated with 500 μM Tyr for 72 hr. GAPDH was used as a loading control (*N* = 3). (b) Cytosolic lysates of H9C2 cells transfected with siScr or siMAO‐A and treated with Tyr for 72 hr were immunoprecipitated with anti‐parkin, anti‐p53 or control IgG antibodies and immunoblotted with parkin and p53 antibodies (*N* = 3). (c) Analysis of p53 by immunoblot in cells transfected with SiScr or Sip53 siRNA for 48 hr. Actin was used as a loading control (representative image of *N* = 3). (d) Analysis of parkin by immunoblot in mitochondrial extracts of H9C2 cells transfected with SiScr or Sip53 siRNA and stimulated with 500 μM Tyr for 72 hr. VDAC was used as a loading control (*N* = 3). (e) Analysis of LC3 and p62 by immunoblot in mitochondrial extracts of cells transfected with siScr or sip53 siRNA and stimulated with 500 μM Tyr for 72 hr. VDAC was used as a loading control (*N* = 3). (f) Analysis of p70S6K phosphorylation in Tyr‐treated cells for 72 hr in the presence or absence of 100 nM rapamycin (rapa) (*N* = 3). (g) Analysis of p70S6K phosphorylation in cells transfected with siScr or siMAO‐A and treated with Tyr for 72 hr (*N* = 4) (h) Analysis of p53 in cytosolic extracts of cells stimulated with 500 μM Tyr for 72 hr in the presence of rapa, when indicated (*N* = 3). GAPDH was used as a loading control. Data are expressed as the mean ± *SEM* (*p < 0.05, **p < 0.01, ***p < 0.001)

Next, we investigated the underlying mechanisms by which increased MAO‐A activation led to impaired mitophagy. It is known that parkin translocation can be inhibited by interaction with cytosolic p53 (Hoshino et al., 2013). In H9C2 cells, we observed that Tyr treatment for 72 hr induced the accumulation of cytosolic p53 levels and increased p53‐parkin interaction (Figure 5a,b). This specific interaction of p53‐parkin induced by Tyr was decreased in cells following siRNA‐mediated knockdown of MAO‐A, confirming the specific role of MAO‐A in this process (Figure 5b). To assess the role of p53 in parkin‐mediated mitophagy, we used p53‐mediated siRNA silencing (Figure 5c). Interestingly, we found that siRNA p53‐transfected cells showed higher levels of mitochondrial parkin than siRNA Scr‐transfected cells after Tyr treatment (Figure 5d). Additionally, mitochondrial levels of LC3II and p62 were strongly increased by Tyr treatment when p53 was silenced, indicative of enhanced mitophagy (Figure 5e). These results suggest that the accumulation of p53 impairs parkin‐mediated mitophagy in senescent cells.

**Figure 6:**
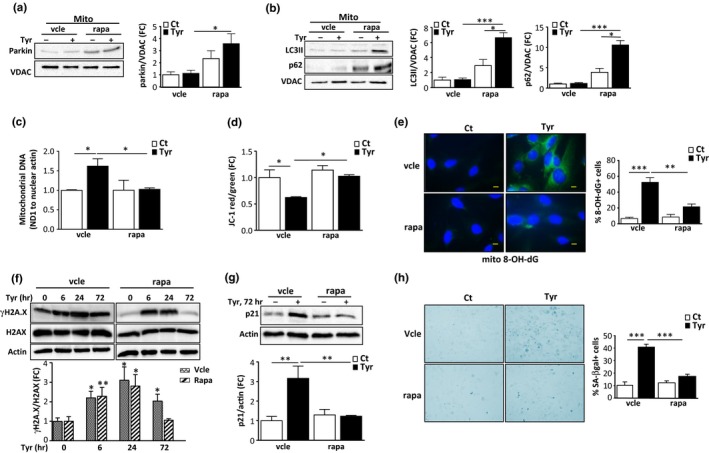
Rapamycin treatment restores mitophagy, preventing the mitochondrial dysfunction, DNA damage response (DDR) and senescence induced by monoamine oxidase‐A (MAO‐A). H9C2 cells were pretreated with vehicle (vcle) or rapamycin (rapa, 100 nM) and stimulated with 500 μM Tyr for 72 hr. (a) Analysis of parkin by immunoblot in mitochondrial extracts; VDAC was used as a loading control for mitochondria (*N* = 3). (b) LC3 and p62 expression in mitochondrial fractions of cells; VDAC was used as a loading control (*N* = 3). (c) mtDNA copy number (ND1/actin) by real‐time PCR (*N* = 3). (d) Quantitative analysis of JC‐1 aggregates (red)/monomer (green) ratios for mitochondrial membrane potential (*N* = 3). (e) Representative images of mito8‐OH‐dG immunostaining and quantitative analysis of 8‐OH‐dG^+^ cells as % of total cells (*N* = 3). (f) Analysis of total and γH2A.X by immunoblot in cells stimulated with Tyr for the indicated times. Actin was used as a loading control (*N* = 3). (g) Analysis of p21 by immunoblot. Actin was used as a loading control (*N* = 3). (h) Representative images and quantitative analysis of SA‐β‐gal^+^ cells as % of total cells after stimulation of the cells with Tyr for 1 week (*N* = 3). Data are expressed as the mean ± *SEM*. (*p < 0.05, **p < 0.01, ***p < 0.001)

Another major regulator of mitochondrial homeostasis and senescence is the protein kinase mTOR, which has been demonstrated to regulate p53 accumulation through MDM2‐dependent or independent mechanisms (Lai et al., 2010). In cells stimulated with Tyr, mTOR was persistently activated, as shown by the phosphorylation level of its major target p70S6K, which was prevented by treatment with the mTOR inhibitor rapamycin (Figure 5f) or by siRNA‐mediated silencing of MAO‐A (Figure 5g). Since rapamycin treatment also inhibited Tyr‐induced p53 accumulation in the cytosol (Figure 5h), we evaluated whether mTOR inhibition impacted mitophagy. As shown in Figure 6a,b, we found that inhibition of mTOR with rapamycin restored the translocation of parkin to the mitochondria in the presence of Tyr and stimulated mitophagy, as shown by the mitochondrial accumulation of LC3II and p62 (Figure 6a,b and Figure S4f). As a consequence of amelioration of mitophagy, rapamycin also prevented the mitochondrial dysfunction induced by Tyr treatment (Figure 6c–e and Figure S4g), and induction of γH2A.X at 72 hr (Figure 6f) and senescent markers p21 and SA‐β‐gal (Figure 6g,h). Altogether, these data show that mTOR is a master regulator of p53‐parkin‐mediated mitophagy inhibition and senescence in response to MAO‐A activation.

## 3 DISCUSSION

This study shows for the first time that MAO‐A can drive SIPS in cardiac cells through ROS production, mitochondrial dysfunction and parkin‐mediated mitophagy inhibition.

MAO‐A‐driven H_2_O_2_ production has been previously assigned to different types of cellular responses, such as hypertrophy and death (Kaludercic et al., 2014). In addition, elevated MAO‐A expression in prostate cancer cells was recently shown to promote metastasis by two distinct mechanisms—epithelial‐to‐mesenchymal transition and paracrine Shh signalling (Wu et al., 2014, 2017 ). These particular responses might depend on the cell type and on the amount of H_2_O_2_, which is known to drive dose‐dependent cellular effects (Duan, Duan, Zhang, & Tong, 2005; Giorgio et al., 2007). During cardiac ischaemia‐reperfusion injury, where high levels of MAO substrates are released, and in the hearts of transgenic mice overexpressing MAO‐A, cell apoptosis and necrosis are preferentially activated together with the lysosomal alteration‐induced blockade of the autophagic flux (Bianchi et al., 2005; Santin et al., 2016; Villeneuve et al., 2013). Here, we demonstrate that chronic sublethal doses of H_2_O_2_ produced by MAO‐A in response to its endogenous (NE) or exogenous (Tyr) substrates can recapitulate all the features of senescence. This could have particular relevance in cardiac ageing, where MAO‐A is likely to be chronically activated due to: (a) sympathetic activation and enhanced release of norepinephrine from adrenergic nerves (Santulli & Iaccarino, 2016); and (b) overexpression of MAO‐A in the ageing heart (as shown in Figure 1a,b) or in age‐associated cardiac diseases (Manni et al., 2016; Villeneuve et al., 2013). In addition, the role of MAO‐A in senescence could be extended to different cell types, since MAO‐A has been characterized in many proliferative cells, such as fibroblasts and bone marrow mesenchymal stem cells, where its expression was significantly increased during ageing (Edelstein & Breakefield, 1986; Trouche et al., 2010).

Cell senescence and mitochondrial dysfunction are considered to be essential “hallmarks of ageing,” and our data provide additional evidence that they are closely interlinked (Lopez‐Otin et al., 2013). Indeed, we observed that mitochondrial dysfunction was necessary for the establishment of the senescent phenotype in response to chronic MAO‐A stimulation. Restoration of mitophagy by overexpression of parkin prevented the persistent activation of the DDR and establishment of senescence. In our model, maintenance of the DDR is tightly controlled by the mitochondria through an amplification loop involving ROS. Parkin overexpression inhibited mitochondrial ROS accumulation and mitochondrial DNA oxidation (8‐OH‐dG), the latter being strongly associated with the ageing process (Barja & Herrero, 2000). Thus, mitochondrial dysfunction through ROS accumulation likely contributes to DDR establishment and senescence. In a previous study, Correia‐Melo et al. (2016) reported that mitochondria were necessary for replicative and irradiation‐induced premature senescence in fibroblasts. In this case, they also observed mitochondrial ROS accumulation and increased mitochondrial mass, but the main drivers of senescence were mitochondrial biogenesis and PGC‐1β activation. This is different from the present study where we show that accumulation of mitochondria is mostly due to the inhibition of mitophagy. However, we cannot exclude the participation of mitochondrial biogenesis, as we showed enhanced levels of PGC‐1β mRNA after MAO‐A activation with Tyr. It is possible that enhanced biogenesis in a situation of mitophagy blockade increases the burden of mitochondrial damage, further contributing to ROS‐DDR and senescence. In addition to its role in mitophagy, parkin has also been demonstrated to act as an inhibitor of the transcriptional repressor PARIS, in turn impacting mitochondrial biogenesis and turnover via regulation of PGC1α and TFEB (Shin et al., 2011; Siddiqui et al., 2015). However, PGC1α mRNA expression was not decreased following MAO‐A activation, and global autophagy was not impaired (Figure S3d). In addition, while the transcription factor TFEB is known to be regulated by mTOR (Roczniak‐Ferguson et al., 2012), its overexpression through TFEB‐adenovirus‐mediated transduction did not prevent senescence induced by MAO‐A (data not shown). Therefore, this transcriptional pathway of parkin‐PARIS‐mediated PGC1α and TFEB regulation does not seem to be operative in this context of senescence induced by MAO‐A.

While we found that mitochondrial dysfunction participated in DDR maintenance, it is also possible that the DDR contributes to mitochondrial dysfunction. Our main results showed that p53, which was under the regulation of the DDR pathway, inhibited mitophagy through its direct interaction with parkin in the cytosol. Consistently, p53 knockdown prevented the decrease in mitochondrial membrane potential and inhibited mitochondrial ROS accumulation. Our results are in line with previous studies that show that the p53‐parkin inhibition pathway plays an important role in cardiac ageing and drives age‐related diseases, such as heart failure and diabetes (Hoshino et al., 2014, 2013 ). Notably, we provide additional mechanistic evidence that p53 is a major mediator of the MAO‐A‐dependent response. Being at the hub of many signalling pathways, we hypothesize that, depending on different stress conditions, p53 can mediate either MAO‐A/H_2_O_2_‐dependent senescence or cell death, as already demonstrated for other stress‐driven mechanisms (Green & Kroemer, 2009). In addition, we cannot exclude the possibility of a direct mechanism of inhibition of parkin through oxidation by H_2_O_2_ as was previously reported (Winklhofer, Henn, Kay‐Jackson, Heller, & Tatzelt, 2003), which would eventually act in cooperation with p53.

Looking for regulatory pathways able to regulate mitochondrial homeostasis, we observed that the mTORC1 kinase complex was potently activated in Tyr‐stimulated cells. There have been many reports of aberrant activation of mTOR during ageing, and inhibition of the mTOR pathway has been shown to extend lifespan and delay age‐associated disorders in various animal models. mTOR can be activated by the DDR through the ATM‐Akt signalling (Correia‐Melo et al., 2016). In the present study, we did not evaluate this possibility, knowing that mTOR can also be activated by many distinct pathways, or directly by ROS (Sarbassov & Sabatini, 2005). We thus tried to understand the consequences of mTOR activation on mitochondrial homeostasis. The mTORC1 complex is well known to inhibit global autophagy through phosphorylation of the initiation complex ULK1 (Nacarelli et al., 2015). However, global autophagy was not modified after MAO‐A stimulation, with no differences in LC3II or p62 levels. Indeed, the regulation of the autophagy machinery during senescence is contradictory, some studies showing higher levels and other showing lower levels of autophagic markers. Conversely, a substantial impairment of mitophagy was consistently observed (Korolchuk et al., 2017). While mTOR is clearly involved in the regulation of mitochondrial homeostasis and mitochondrial biogenesis (Morita et al., 2013), its regulatory role in mitophagy has been underestimated. Here, we provide evidence that rapamycin, an inhibitor of the mTORC1 complex, completely restores mitophagy and prevents all the features associated with mitochondrial dysfunction, DDR and senescence. In addition, we describe for the first time the interaction between two major senescence‐associated pathways, mTOR and p53, which cooperate to inhibit mitophagy and stabilize the DDR and senescence in response to MAO‐A (Figure S5) (Nacarelli et al., 2015).

Finally, it is possible that MAO‐A‐driven SIPS may act as a deleterious mechanism, enhancing the susceptibility of the elderly to cardiac diseases. This will need to be evaluated in future studies, together with the possibility that MAO‐A inhibition may prevent or reduce cardiac pathological ageing.

## 4 EXPERIMENTAL PROCEDURES

### 4.1 Materials

Tyramine, norepinephrine, clorgyline, Trolox and rapamycin were from Sigma‐Aldrich (St. Louis, MO, USA). The 3X‐HA‐3X‐Flag‐hparkin insert was subcloned from pcDNA5/FRT/TO into the pcDNA3 plasmid (pcDNA5/FRT/TO was a generous gift from Lars Dreir, Department of Neurobiology at UCLA, Los Angeles, USA). The pEGFP‐LC3 plasmid was from Adgene.

### 4.2 Cell culture

Adult ventricular myocytes were obtained from hearts of male C57Bl6J mice at 3 and 20 months, as previously described (Fazal et al., 2017). All animal procedures conformed to the Guide for the Care and Use of Laboratory Animals published by the Directive 2010/63/EU of the European Parliament. Rat H9c2 cardiomyoblasts (American Type Culture Collection, Rockville, USA) were grown in DMEM media containing 10% heat‐inactivated FBS under 5% CO_2%_ and 95% air at 37°C. H9C2 served as an animal‐free alternative, sharing many physiological properties of primary cardiac cells (Watkins, Borthwick, & Arthur, 2011). Cells were transfected with MAO‐A siRNA, p53 siRNA or scramble siRNA (on‐target‐plus smart pool; Dharmacon) with DharmaFECT Duo (Dharmacon). Plasmid transfections were performed with Lipofectamine (Thermo Fisher Scientific). Neonatal rat ventricular myocytes (NRVMs) were obtained from neonatal rats 1–2 days old. The heart was excised and the atria were removed. Primary cultures of NRVMs were subsequently performed, as previously described (Santin et al., 2016). NRVMs were transduced with an adenovirus expressing rat MAO‐A under the control of the CMV promoter to drive expression of MAO‐A. After 24 hr, the medium was replaced with Ham‐F12 medium supplemented with FBS, and the pharmacological treatments were performed.

### 4.3 Subcellular fractioning and Western blot analysis

Mitochondrial and cytosolic fractions were isolated using a commercial kit (Qiagen, Germany). For whole‐cell lysates, cells were collected in RIPA buffer (20 mM Tris‐HCl, 150 mM NaCl, 1 mM EDTA, 1% NP40, 0.1% SDS, antiphosphates and antiproteases) and centrifuged at 13,000 *g* for 5 min to obtain the supernatant. Equal amounts of proteins were electrophoresed and transferred to a nitrocellulose membrane. Primary antibody incubations were performed with anti‐MAO‐A or anti‐parkin from Abcam; anti‐p53, anti‐phospho‐p53(ser15), anti‐H2A.X, anti‐pink1, anti‐LC3, anti‐ubiquitin, anti‐phospho‐Rb(Ser807/811), anti‐phospho‐p70S6K(Thr389) from Cell Signaling Technologies; anti‐phospho‐H2A.X(Ser139) from Millipore; anti‐p62 from Abnova; and anti‐ATM, anti‐phospho‐ATM(Ser1981), anti‐p21 from Santa Cruz Biotechnology. Images were taken with the ChemiDoc‐MP Imaging System and quantified using Image‐Lab 4.0 software (Bio‐Rad).

### 4.4 Immunoprecipitation assay

For immunoprecipitation (IP), the cytosolic fractions were incubated with anti‐p53 or anti‐parkin antibodies for 12 hr at 4°C. Protein A/G Plus‐Agarose was then added for 3 hr at 4°C on a rotating device. Immunoprecipitates were collected by centrifugation at 6,000 *g* at 4°C and washed with lysis buffer (20 mM Tris pH 7.5, 150 mM NaCl, 1 mM EDTA, 1% Triton X‐100, proteases and phosphatase inhibitors). The pellets were eluted by heating at 95°C for 5 min in electrophoresis sample buffer and subjected to immunoblotting.

### 4.5 Immunofluorescence

H9C2 cells were fixed with 4% PFA, permeabilized with 0.5% Triton, blocked with 3% BSA and incubated with anti‐vinculin (Sigma) or anti‐pH2A.X (Millipore) overnight at 4°C. The secondary antibody was Alexa‐Green‐488 goat anti‐mouse (Invitrogen). Images were acquired using epifluorescence microscopy (DM600 microscope; Leica). For mitophagy detection, EGFP‐LC3 plasmid and MitoID (Enzo) were used to stain autophagosomes and mitochondria, respectively. Image acquisition was performed with an LSM780 laser scanning confocal microscope (Carl Zeiss). For mitochondrial 8‐OH‐dG detection, cells were fixed with methanol for 30 min at −20°C, permeabilized with 0.2% Triton and treated with RNase A at 37°C for 1 hr, followed by denaturation with ice‐cold 25 mM NaOH in 50% ethanol, as previously described (Ohno, Oka, & Nakabeppu, 2009). After blocking with 10% BSA, the cells were incubated with mouse anti‐8‐OH‐dG (clone 483.15; Millipore) overnight at 4°C. The secondary antibody was goat anti‐mouse Alexa 488.

### 4.6 Comet assay

DNA breaks were measured with the comet assay, as previously described (Collins et al., 2008). The cell suspensions were mixed with 1% low melting point agarose, and the mixture was spread onto slides that were precoated with 1% normal melting point agarose (Sigma). Glass cover slips were placed on the drops of agarose, which were allowed to set at 4°C. The cover slips were then removed and the cells that were embedded in agarose were lysed for 1 hr by immersion in lysis solution (2.5 M NaCl, 0.1 M Na_2_EDTA, 0.1 M Tris base, pH 10% and 1% Triton X‐100) at 4°C. The slides were then placed in a horizontal gel electrophoresis tank and the DNA was allowed to unwind for 40 min in freshly prepared alkaline electrophoresis solution (0.3 M NaOH and 1 mM Na_2_EDTA, pH > 13). Electrophoresis was carried out in the alkaline solution for 30 min at 4°C. The slides were washed in 0.4 M Tris base (pH 7.5) for 10 min at 4°C to neutralize the excess alkali, followed by 10 min in water at 4°C. They were then left to dry overnight. The gels were stained with 25 µl of DAPI, covered with a cover slip and coded prior to microscopic analysis. DAPI‐stained nuclei were evaluated with a fluorescence microscope.

### 4.7 Total ROS

Cellular ROS were measured using the fluorescent probe DCFDA assay at a concentration of 5 µM (Thermo Fisher Scientific).

### 4.8 *SA‐*β*‐gal staining*

SA‐β‐gal activity was measured as previously described (Duan et al., 2005). Cells fixed in PFA were stained with SA‐β‐gal stain solution (1 mg/ml X‐gal, 40 mM citric acid/sodium phosphate, pH 6, 5 mM potassium ferrocyanide, 5 mM potassium ferricyanide, 150 mM NaCl, 2 mM MgCl_2_). After 16 hr at 37°C, images were taken using a brightfield microscope.

### 4.9 JC1 staining

The mitochondrial membrane potential was evaluated by JC1 probe (Thermo Fisher Scientific). Before the end of the treatments, cells were loaded with JC1 probe at a concentration of 5 µg/ml for 15 min and analysed for imaging with a confocal microscope.

### 4.10 Real‐time RT‐PCR

The extraction of RNA from H9C2 cells was performed using column affinity purification (Qiagen). cDNAs were synthesized using the iScript RT master mix (Bio‐Rad) with random hexamers. Real‐time PCR was performed on a StepOnePlus system (Applied Biosystems) in 96‐well plates with specific primers and SYBR green mix (Bio‐Rad). The rat primers were as follows: PGC‐1α‐F: CACCAAACCCACAGAGAACAG, PGC‐1α‐R: GCAGTTCCAGAGAGTTCCACA; PGC1‐β‐F: TTGTGTCAAGGTGGATGGCA and PGC‐1β‐R: GCACCGAAGTGAGGTGCTTA; p21‐F: TGCCGAAGTCAGTTCCTTGT and p21‐R: GTTCTGACATGGCGCCTCC; p16‐F: CTTCGGCTGACTGGCTGG and p16‐R: TCATCATGACCTGGATCGGC; p15‐F: GGGACTAGTGGAGAAGGTGC and p15‐R: CATCATCATGACCTGGATCGC. GAPDH was used as an endogenous control as follows: GAPDH‐F: TCTCTGCTCCTCCCTGTTCTA and GAPDH‐R: TCCGATACGGCCAAATCCGTT. The relative mRNA expression levels were calculated by applying the following equation: 2^−∆^ *^C^* ^t^, and the fold‐change value of expression compared to control was calculated following the ΔΔ*C*_t_ method.

### 4.11 mtDNA copy number

The mtDNA copy number was evaluated by real‐time PCR on extracted DNA, with specific primers for mitochondrial (ND1‐F: ATGGATTCGAGCATCCTACCC, ND1‐R: TCCTGCTAGGAAAATTGGCA; CytB‐F: TGCCGAGACGTAAACTACGG, CytB‐R: TAGTCCTCGTCCCACATGGA) and nuclear gene (β‐actin‐F: GCAGGAGTACGATGAGTCCG, β‐actin‐R: ACGCAGCTCAGTAACAGTCC). The mtDNA copy number was normalized to nuclear gene.

### 4.12 Mitochondrial respiration

The oxygen consumption rate (OCR) was measured with a Seahorse XFe24 Analyzer (Agilent). Cells were plated in Seahorse 24‐well assay plates in complete medium, and after 2 hr of cell attachment the medium was replaced with XF base medium supplemented with 10 mM glucose, 4 mM l‐glutamine and 1 mM sodium pyruvate (pH 7.4). The plates were incubated for an additional 1 hr at 37°C in a non‐CO_2_ incubator. The wells of a hydrated sensor cartridge were then loaded with 1 µM oligomycin (port A), 1 µM carbonyl cyanide 4‐(trifluoromethoxy)phenylhydrazone (FCCP) (port B) and 1 µM antimycin A + 1 µM rotenone (port C). Data were analysed using the Seahorse Wave software.

### 4.13 Statistical analysis

Statistical analysis was carried out using Student's *t*‐test or 2‐way ANOVA with the Tukey post hoc test, when appropriate. The results are shown as the mean ± *SEM*. Values of *p* < 0.05 were considered to be significant.

## AUTHOR CONTRIBUTIONS

NM designed, performed and analysed the majority of experiments. YS, DM and HM performed and analysed individual experiments. VDE, CB and JMP designed and supervised individual experiments. FL, CB, AP, JFP and JMP supervised the study and wrote the manuscript, with contributions from all authors.
