# Correction to “Monoamine Oxidase‐A Is a Novel Driver of Stress‐Induced Premature Senescence Through Inhibition of Parkin‐Mediated Mitophagy”

**DOI:** 10.1111/acel.70531

**Published:** 2026-05-08

Manzella, N., Y. Santin, D. Maggiorani, et al. 2018. Monoamine Oxidase‐A Is a Novel Driver of Stress‐Induced Premature Senescence Through Inhibition of Parkin‐Mediated Mitophagy. *Aging Cell* 17:e12811. https://doi.org/10.1111/acel.12811.

(1) An error was made when putting together the representative images for panel 4f and panel 6f during manuscript preparation. A confusion occurred between control conditions (in Figure 1i) and vehicle + empty plasmid (vcle/pcDNA3) conditions (Figures 4f and 6f). The vcle/pcDNA3 is now shown in both Figures 4f and 6f. The authors were able to provide all the original images to the editor.

**FIGURE 4:**
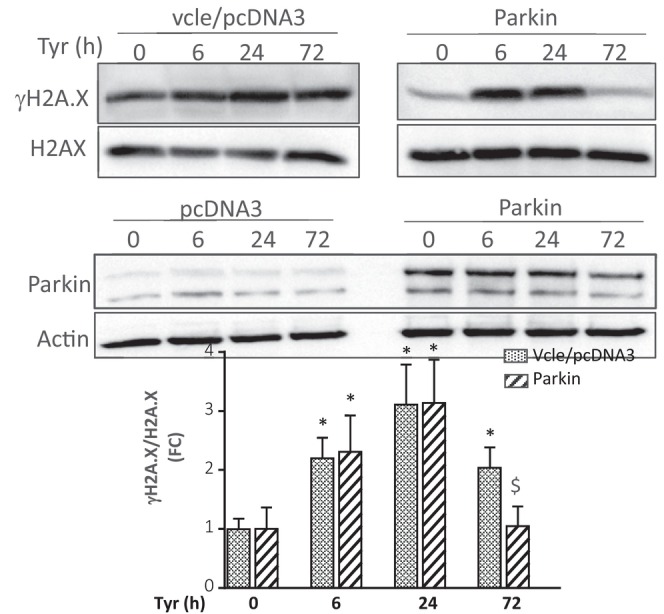
(f) Analysis of γH2A.X by immunoblot in cells stimulated with Tyr for the indicated times and normalized to H2A.X (*N* = 3).

**FIGURE 6:**
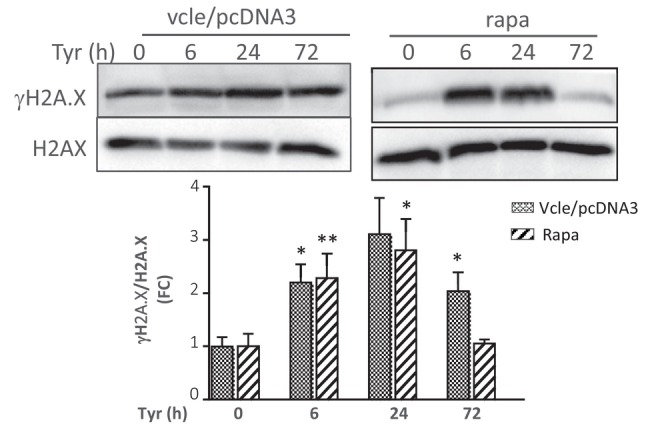
(f) Analysis of γH2A.X by immunoblot in cells stimulated with Tyr for the indicated times and normalized to H2A.X (*N* = 3). The vcle/pcDNA3 control immunoblot is also shown in Figure 4f.

(2) An error was made when putting together the representative images for Figure S2C. The actin immunoblot was added incorrectly and was not used or quantified in the manuscript. Figure S2C has been updated accordingly.

(3) In Figure S4B, the image displayed in the immunoblot was incorrectly incorporated and comes from preliminary experiments to set up the cell transfection protocol. It should not appear in the final article. We were able to provide the good images of final experiments to the editor, with the GAPDH as a loading control. Figure S4B has been updated accordingly.

The authors confirm that all the experimental results and corresponding conclusions mentioned in the paper remain unaffected.

We apologize for these errors.

## Supporting information


**Figure S2C:** acel70531‐sup‐0001‐Figures.docx.
**Figure S4B:** acel70531‐sup‐0001‐Figures.docx.

