# Supplementary figures and images for: Monoamine oxidase‐A is a novel driver of stress‐induced premature senescence through inhibition of parkin‐mediated mitophagy
Source: Aging Cell. 2018 Jul 12;17(5):e12811. doi: 10.1111/acel.12811 (PMC6156293; doi:10.1111/acel.12811)

**A**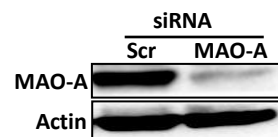**B**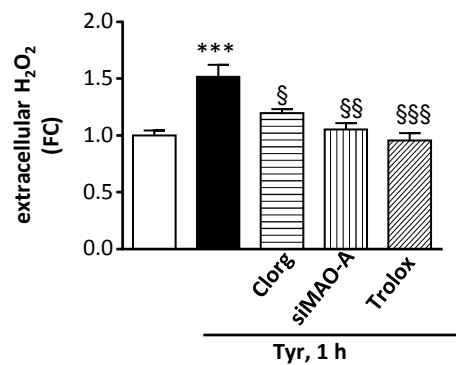**C**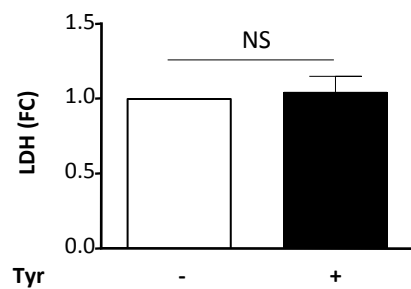**D**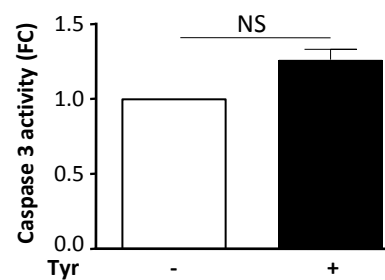**E**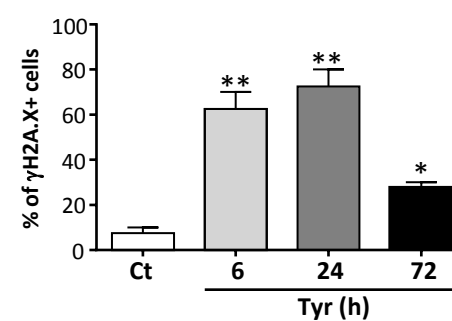**F**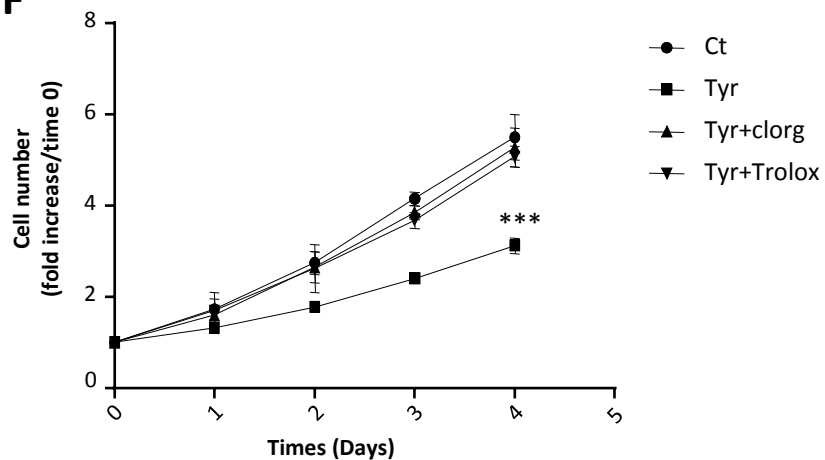

Supplementary Fig 1

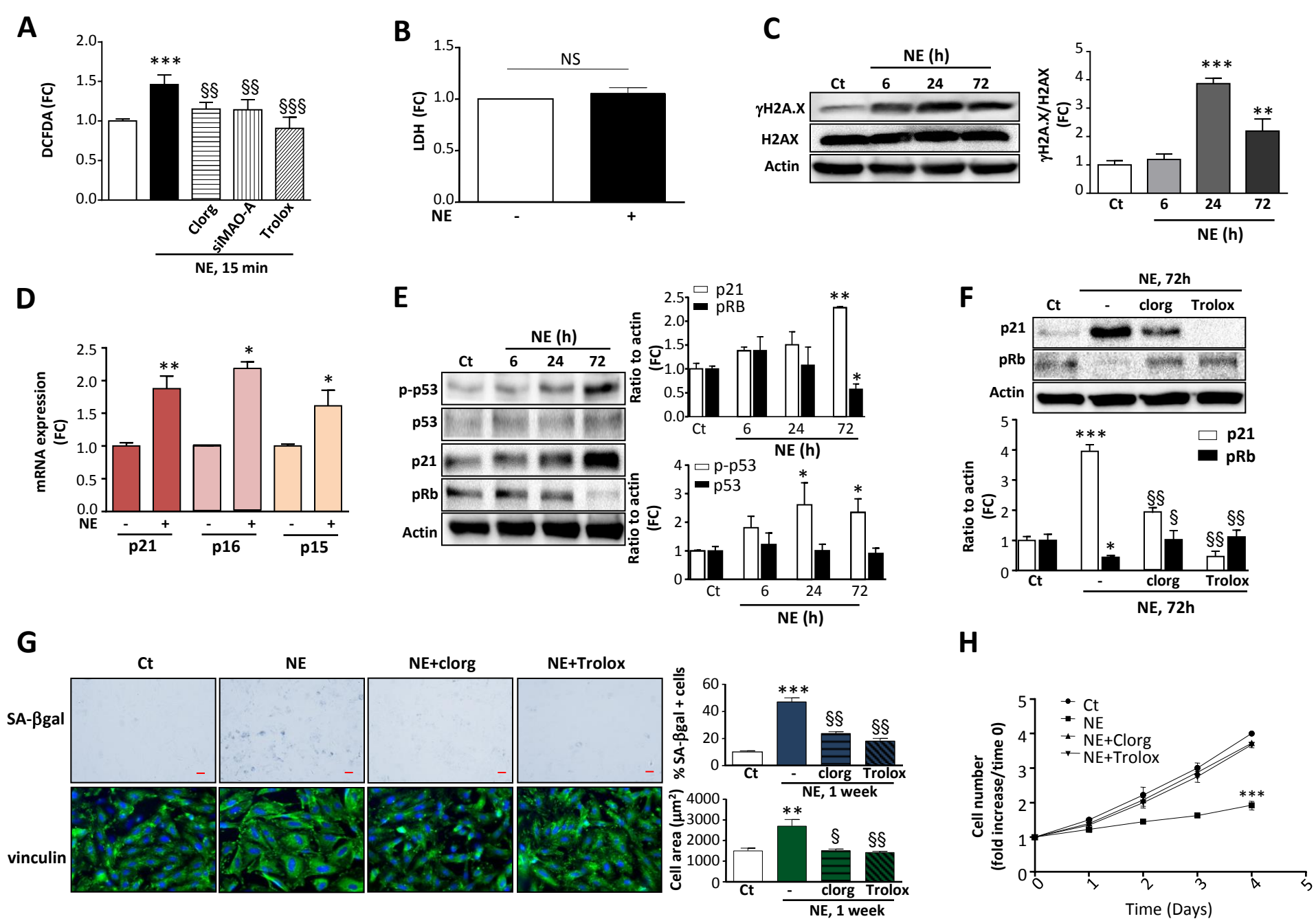

Supplementary Fig 2

**A**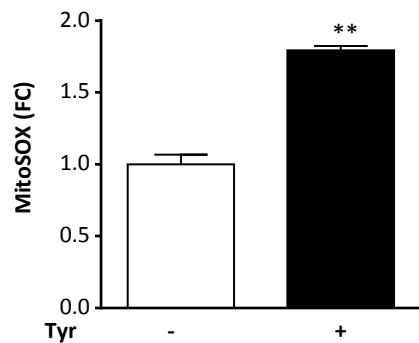**B**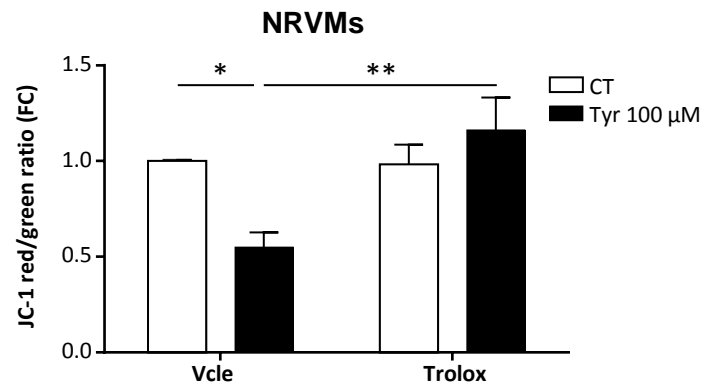**C**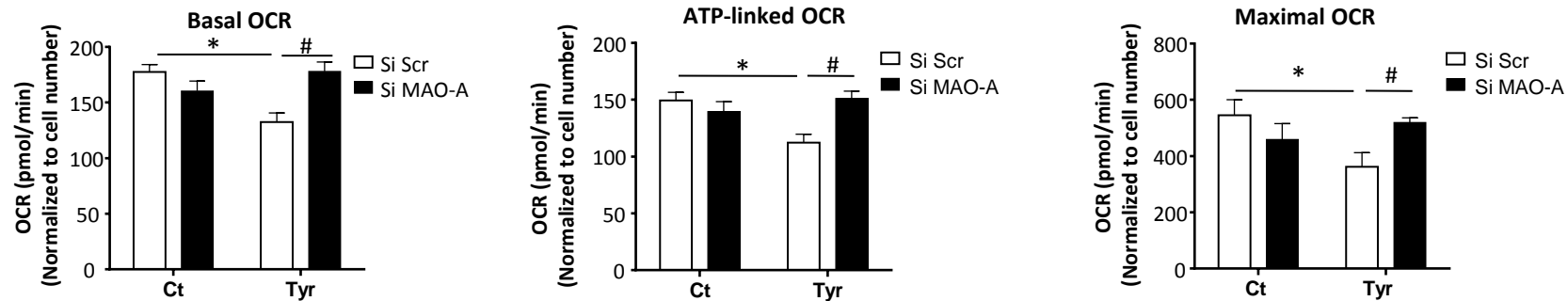**D**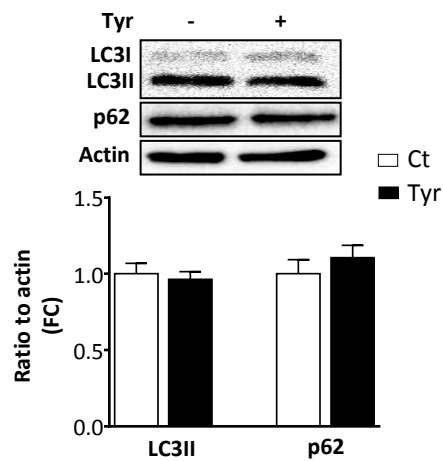**E**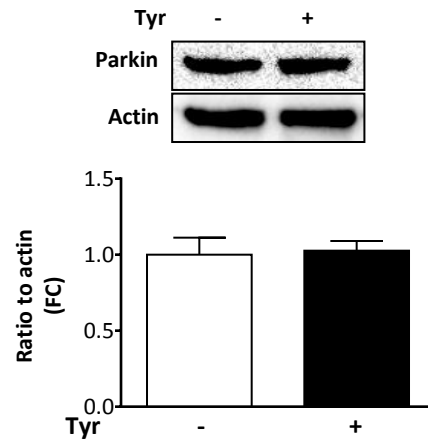

**A**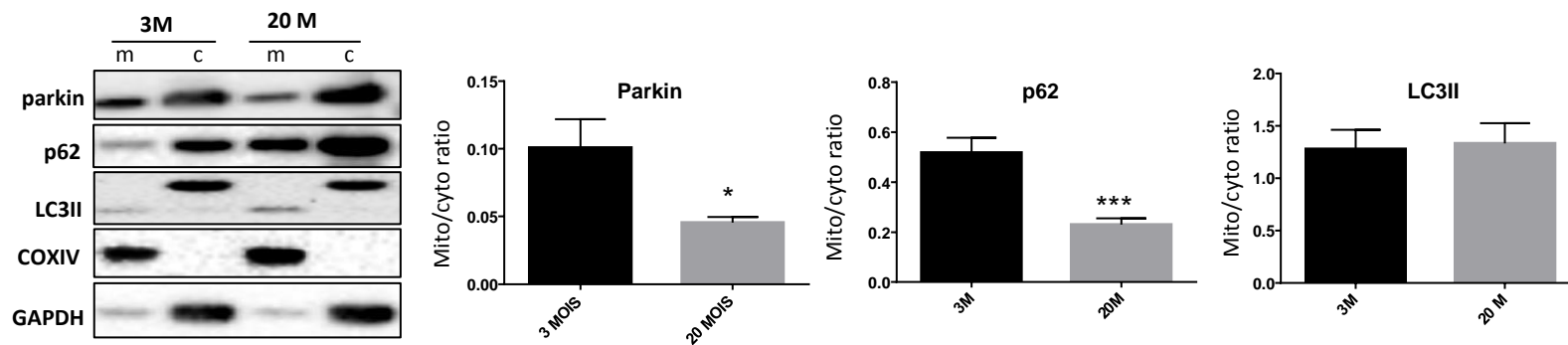**B**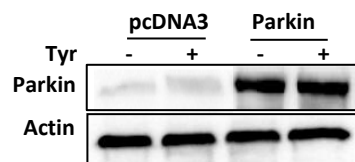**C**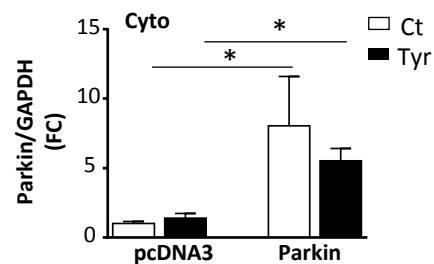**D**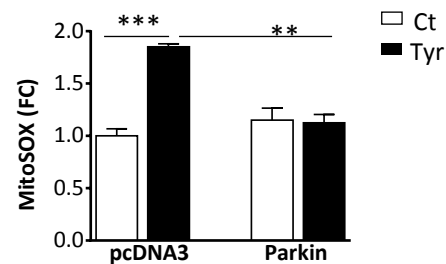**E**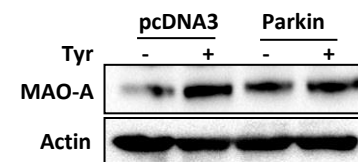**F**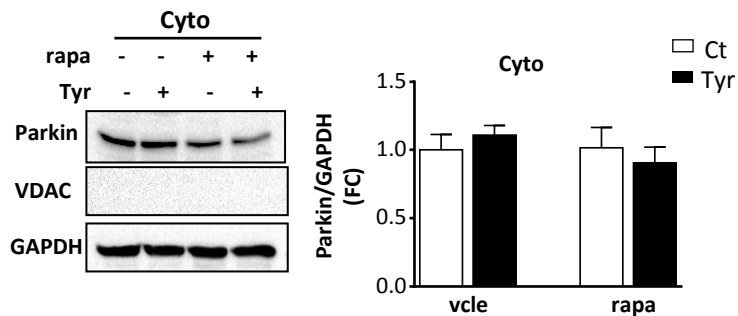**G**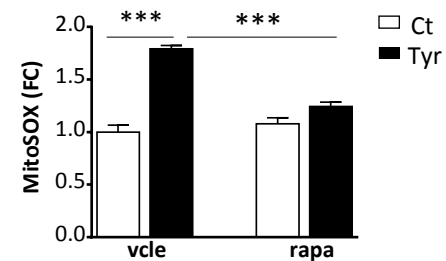

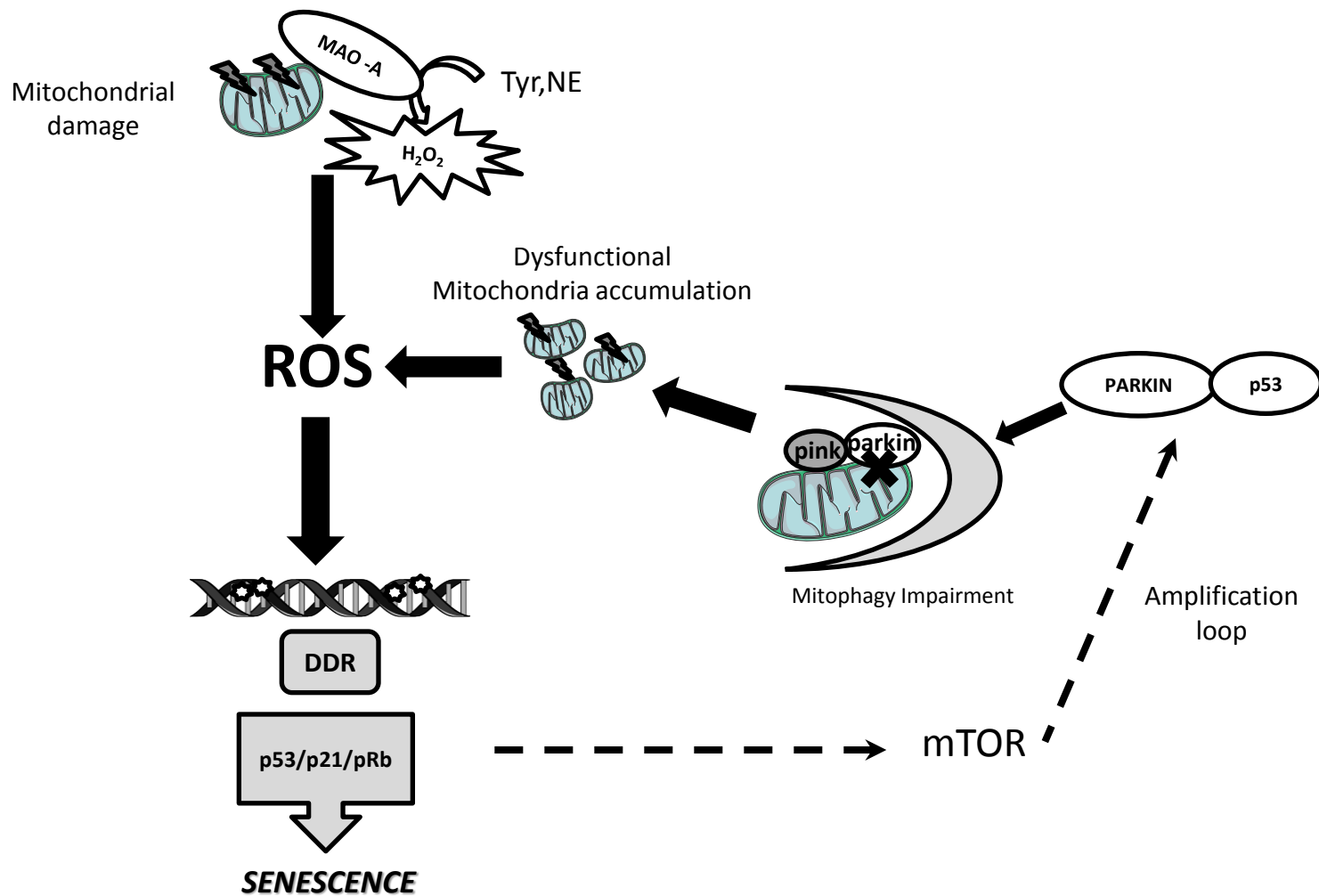

Supplementary Fig 5

Supplement: Supplementary file 1 [file ACEL-17-e12811-s001.pdf]
