# Supplementary material for: Monoamine oxidase‐A is a novel driver of stress‐induced premature senescence through inhibition of parkin‐mediated mitophagy
Source: Aging Cell. 2018 Jul 12;17(5):e12811. doi: 10.1111/acel.12811 (PMC6156293; doi:10.1111/acel.12811)
Supplement: Supplementary file 2 [file ACEL-17-e12811-s002.doc]

**Material and methods**

***Cell proliferation rate***

H9C2 cells were seeded in 6-well plates and various treatments were added to the medium during 1 week. After removal of the medium, the cells were harvested and plated at a density of 5x10^4^/well in fresh culture medium and the proliferation rate was evaluated by counting the cells at indicated times.

***Cell Viability and Cell Death Assay***

For quantitative assessment of necrosis, LDH released in culture medium was measured using LDH cytotoxicity Assay Kit according to the manufacturer’s instructions (Biovision). Apoptosis was measured as caspase-3 activation using a commercial kit (Biotium) according to the manufacturer's instructions.

***Mitochondrial ROS and H_2_O_2_ measurements***

The mitochondrial ROS was measured by mitoSOX probe (Invitrogen, Molecular Probes). Brieﬂy, the cells were loaded with mitoSOX probe at a ﬁnal concentration of 5 µM and incubated for 30 min after the indicated treatments. Cells were resuspended in HBSS before reading to Tecan plate reader. Extracellular H_2_O_2_ detection was evaluated by Amplex-Red probe (Invitrogen, Molecular Probes) on cell supernatants collected and mixed with an Amplex red solution.

**Figure legends**

**Supplementary Figure 1**

**(A)** Immunoblots of MAO-A expression in scramble-siRNA (Scr) or MAO-A-siRNA-transfected cells with actin as a loading control. (N=3). **(B)** H_2_O_2_ measurements in the extracellular media with Amplex Red probe in response to 500 *μ*M Tyr, and in the presence of clorg (10 μM), MAO-A siRNA or Trolox (500 μM), when indicated (N=3). **(C)(D)** Measurements of Lactate Dehydrogenase release in culture media and caspase-3 activity in cells treated with 500 μM Tyr for 24 h (N=3). NS, non statistical. **(E)** Quantification of γH2A.X positive cells as % of total cells after immunostaining with γH2A.X antibody in H9C2 cells treated with Tyr for increasing times (N=3). **(F)** Proliferation rate of H9C2 cells evaluated at indicated times after 7d of Tyr (500 μM) treatment in the presence of clorg (10 μM) or Trolox (500 μM), when specified (N=3). Data are expressed as means ± sem (*p<0.05, **p<0.01, ***p<0.001 vs control; §p<0.05, §§p<0.01, §§§p<0.001 vs Tyr).

**Supplementary Figure 2**

**(A)** DCFDA oxidation in response to 100 *μ*M NE for 15 min, and in the presence of clorg (10 μM), siMAO-A siRNA or Trolox (500 μM), when indicated (N=3). **(B)** Measurements of Lactate Dehydrogenase release in culture media in cells treated with 100 μM NE for 24 h (N=3). NS, non statistical. **(C)** Immunoblots of total and phosphorylated levels of H2A.X in H9C2 cells after stimulation with NE (100 μM) for the indicated times. Actin was used as loading control. Quantifications of the ratios of γH2A.X on total H2A.X are shown on the right panel (N=3). **(D)** Analysis of mRNA levels of CDKi p21^cip^, p16^ink4a^ and p15^ink4b^ normalized to GAPDH by real-time RT-PCR in cells stimulated with 100 μM NE for 72 h (N=5). **(E)** Immunoblots of phospho(Ser15)p53, total p53, p21 and pRb in cells stimulated with 100 μM NE for 6 to 72 h. Quantifications of the ratios to actin are shown on the histograms on the right (N=3). **(F)** Immunoblots of p21 and pRb in cells stimulated with 100 μM NE for 72 h and pretreated with clorg or Trolox, when specified. Quantifications of the ratios to actin are shown on the histogram below (N=3).  **(G)** *upper panel:* representative images and quantitative analysis of SA-β-gal+ cells expressed as % of total cells after stimulation with 100 μM NE for 1 week in the presence of clorg (10 μM) or Trolox (500 μM), when indicated. For each condition, a total of 100 cells were counted, scale Bar= 100 μm (N=3); *Lower panel*: representative images and quantitative analysis of cell area after stimulation with 100 μM NE for 1 week in the presence of clorg (10 μM) or Trolox (500 μM), when indicated. DAPI (blue) was used to label nucleus and Vinculin for cell size measurement (green). Scale Bar= 10 μm. For each condition, a total of 100 cells were counted (N=3). **(H)** Proliferation rate of H9C2 cells evaluated at indicated times after 1 week of treatment with 500 µM Tyr in the presence of clorg (10 μM) or Trolox (500 μM), when specified (N=3). Data are expressed as means ± sem (*p<0.05,**p<0.01, ***p<0.001 vs control; §p<0.05, §§p<0.01 vs Tyr).

**Supplementary Figure 3**

**(A)** Mitochondrial ROS with MitoSOX probe in H9C2 cells treated with 500 μM Tyr for 72h. **(B)** Quantitative analysis of JC-1 aggregates (red)/monomers (green) ratios for mitochondrial membrane potential in neonatal rat ventricular myocytes (NRVMs) stimulated with Tyr 100 μM for 24 h and assessed 72 h later (N=5). **(C)** Quantification of Basal, Maximal and ATP-linked Oxygen Consumption Rates (OCR) in H9C2 cells transfected with siScr or siMAO-A and treated 24 h later with Tyr for 24 h. Recording of respiration was done at baseline and after successive addition of Oligomycin, FCCP and Antimycin A + Rotenone 72 h after Tyr treatment. **(D)** Analysis of LC3 and p62 levels by immunoblot in cells stimulated with 500 μM Tyr for 72h. Actin was used as a loading control (N=3). **(E)** Analysis of Parkin levels by immunoblot in cells stimulated with 500 μM Tyr for 72h. Actin was used as a loading control (N=3). Data are expressed as means ± sem. (***p<0.001).

**Supplementary Figure 4**

**(A)** Immunoblot on Parkin, p62 and LC3II proteins in mitochondrial and cytoplasmic fractions from young (3 months) and old (20 months) mice hearts (N=9-11). **(B)** Analysis of Parkin levels by immunoblot in cells transfected with pcDNA3 or Parkin and stimulated with 500 μM Tyr for 72h. Actin was used as a loading control (N=3). **(C)** Analysis of Parkin levels by immunoblot in cytosolic extracts from cells transfected with pcDNA3 or Parkin and stimulated with 500 μM Tyr for 72h. GAPDH was used as a loading control (N=3). **(D)** Mitochondrial ROS with MitoSOX probe in H9C2 transfected with pcDNA3 or Parkin and treated with 500 μM Tyr for 72h (N=5). **(E)** Analysis of MAO-A levels by immunoblot in cells transfected with pcDNA3 or Parkin and stimulated with 500 μM Tyr for 72h. Actin was used as a loading control (N=3). **(F)** Analysis of Parkin levels by immunoblot in cytosolic extracts from cells pre-treated with rapamycin and stimulated with 500 μM Tyr for 72h. GAPDH was used as a loading control (N=3). **(G)** Mitochondrial ROS with MitoSOX probe in H9C2 pre-treated with rapamycin and stimulated with 500 μM Tyr for 72h (N=5). Data are expressed as means ± sem. (*p<0.05, **p<0.01, ***p<0.001)**.**

**Supplementary Figure 5**

Schematic representation of MAO-A-induced senescence signaling pathway**.** MAO-A-induced H_2_O_2_ generation causes mitochondrial oxidative damage and ROS generation leading to DDR and activation of the p53/p21 pathway. Accumulation of p53 in the cytoplasm through mTOR activation impairs mitochondrial quality control, leading to further accumulation of damaged mitochondria within an amplification loop that stabilizes DDR and senescence.
