# Supplementary material for: Correction to “Monoamine Oxidase‐A Is a Novel Driver of Stress‐Induced Premature Senescence Through Inhibition of Parkin‐Mediated Mitophagy”
Source: Aging Cell. 2026 May 8;25(5):e70531. doi: 10.1111/acel.70531 (PMC13154755; doi:10.1111/acel.70531)
Supplement: Supplementary file 1 — Figure S2C: acel70531‐sup‐0001‐Figures.docx. Figure S4B: acel70531‐sup‐0001‐Figures.docx. [file ACEL-25-e70531-s001.docx]

Corrected Figure S2C:

**Ct 6 24 72**

**NE (h)**

0

1

2

3

4

5

***

**

**γH2A.X/H2AX**

**(FC)**


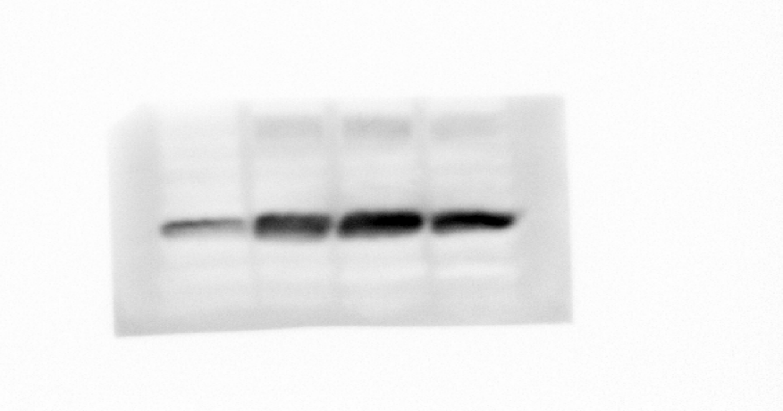

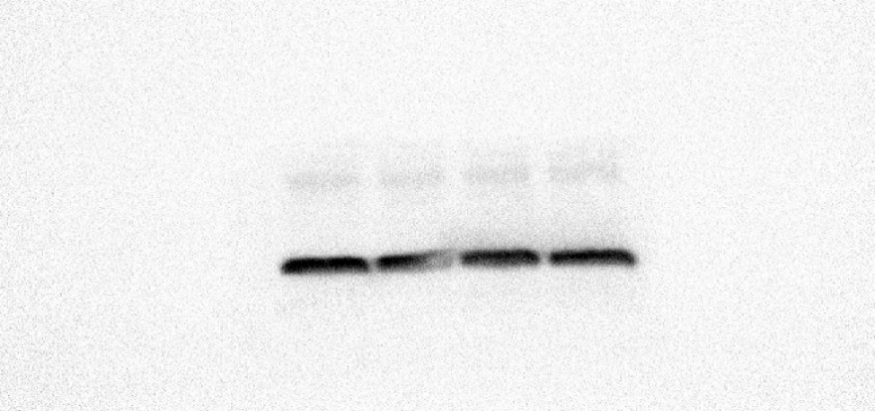


NE (hr)

0 6 24 72

γH2A.X

H2A.X

Corrected Figure S4B:


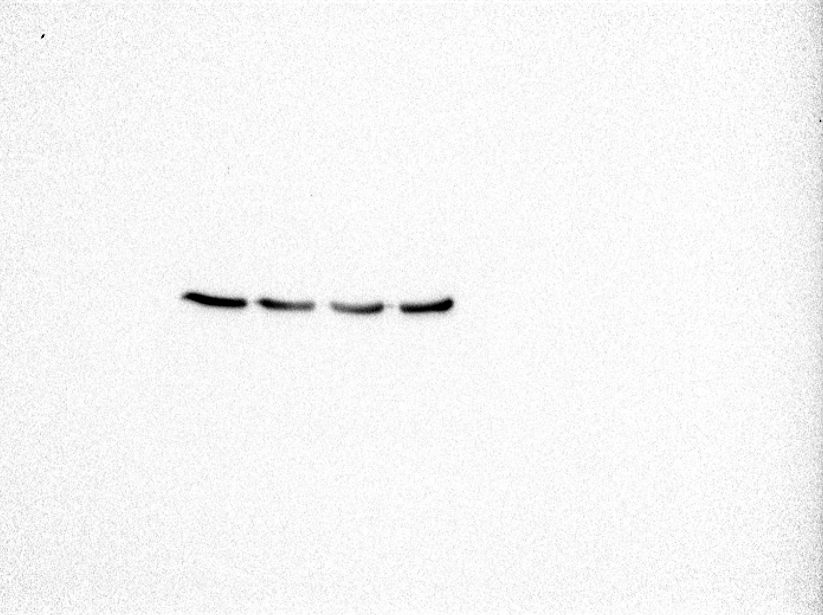


Tyr - + - +

pcDNA3

Parkin

Parkin

GAPDH


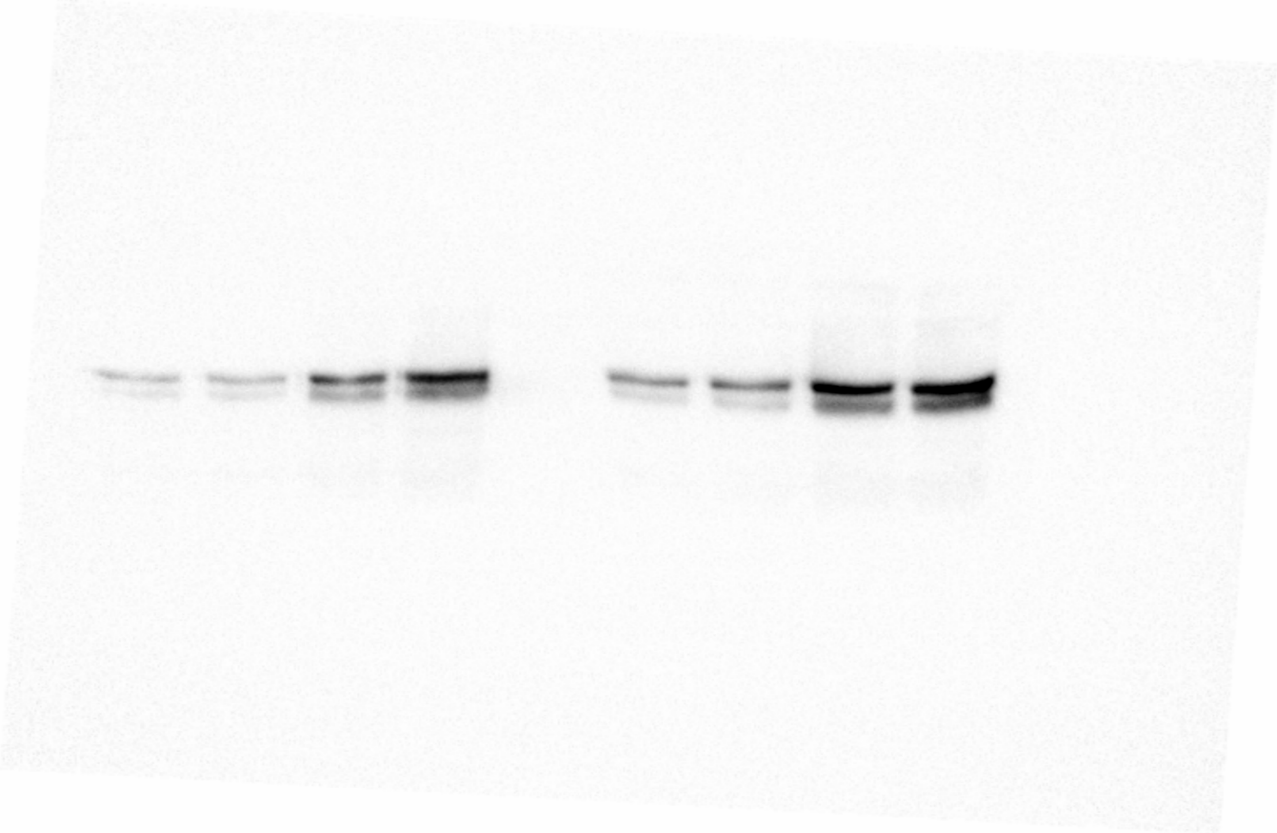


**Cytosol**
